# Development and evaluation of a gamified smart phone mobile health application for oral health promotion in early childhood: a randomized controlled trial

**DOI:** 10.1186/s12903-020-01374-2

**Published:** 2021-01-07

**Authors:** Mitra Zolfaghari, Mina Shirmohammadi, Houra Shahhosseini, Mehrshad Mokhtaran, Simin Z. Mohebbi

**Affiliations:** 1 Department of E-Learning in Medical Education, Virtual School, and Nursing and Midwifery Care Research Center, School of Nursing and Midwifery, Tehran University of Medical Sciences, Tehran, Iran; 2 Research Centre for Caries Prevention, Dentistry Research Institute, Tehran University of Medical Sciences, P.O.Box 1439955991, Tehran, Iran; 3 Department of Community Oral Health, School of Dentistry, Tehran University of Medical Sciences, Tehran, Iran; 4 Qazvin University of Medical Sciences, Qazvin, Iran; 5 Department of E-learning in Medical Education, Virtual School, Tehran University of Medical Sciences, Tehran, Iran

**Keywords:** Application, Gamification, Mother, Oral health, Preschooler

## Abstract

**Background:**

This study aimed to design a gamified smartphone application (app) and assess its efficacy for education of mothers regarding oral healthcare of their children.

**Methods:**

In this pretest–posttest controlled clinical trial, a simple app and a gamified version of it were designed to enhance the oral health knowledge and practice of mothers. The app contains information about early childhood caries, health diet, sugars, baby-oral hygiene, fluoride effect, fluoride toothpaste, tooth-brushing training video and regular dental visits. The opinion of experts and 3 mothers were obtained and both apps were revised accordingly. The intervention was implemented on mothers of preschoolers referring to the specialty dental clinic of Tehran School of Dentistry in 2019. The mothers were randomly allocated to the simple app or gamified app group. Before the intervention, all mothers filled out a questionnaire regarding oral health knowledge and practice, and their demographics were collected. The plaque index (PI) of children was also measured. The mothers filled out the same questionnaire 1 month after the intervention, and the PI of children was measured again. Paired *t* test and linear regression model were used for statistical analysis of the data.

**Results:**

Totally, 58 mother and child pairs entered the study; 40% of children were boys. The mean age of children was 4.7 ± 1.2 years. The mean knowledge score of mothers in the pretest was 10.5 and 11.3 in simple app and gamified app group, respectively, which changed to 13.1 and 14.3, respectively in the posttest. The mean practice score of mothers was 4.4 and 4.8 in simple app and gamified app groups, respectively in the pretest, which changed to 8.5 and 8, respectively in the posttest. The mean dental plaque index of children in the pretest was 0.8 and 1 in simple app and gamified app groups, respectively, which changed to 0.5 and 0.5, respectively in the posttest. Children had better Plaque control in gamified app group (*P* < 0.05).

**Conclusion:**

After 1 month, both apps effectively improved the oral-health knowledge and practice of mothers while oral hygiene as a result of plaque control was superior in children of mothers using the gamified app.

*Trial registration* IRCT, IRCT20131102015238N2. Registered 24 February 2019—Retrospectively registered, https://fa.irct.ir/trial/36600.

## Background

Mobile health is a new term that refers to the use of mobile phones and wireless communication technologies to promote healthcare. This technology enhances tele-medicine, data collection (for application in community health or clinical purposes) and decision making, provides simple and fast access to healthcare information, promotes a fast response system in emergency situations, helps to control patient condition, and improves patient compliance to treatment [1].

Application of smartphones has greatly increased in the recent years. In 2018, one-third of the world’s population had smartphones [2]. The increasing use of smartphones led to explosive growth in usage of smartphone applications (apps) aiming to promote the health status and hygienic behaviors, In 2019, a systematic search in app stores found 612 apps related to oral health [3]. Another systematic review showed that Mobile-health strategies could work as additional tool for enhancing oneself oral hygiene, in particular to manage gingivitis, and to improve oral health knowledge [4]. In addition, some apps are designed to influence parents' attitudes, beliefs, and behaviors towards their children’s oral health [5].

People are interested to have their health information readily available in their smartphones. At present, over 500 million people worldwide use health-related smartphone apps [6]. These apps have a simple performance and only provide simple information [7]. The opinion of the experts regarding general health and behavioral change has not been asked in the process of manufacturing of many of these apps and they have been simply designed based on the preferences of the users. Thus, they have limited advantage in behavioral change [7–10]. Therefore, further investigations are required on the advantages and potential disadvantages of such apps [10]. Apple store and Google Play store only assess the apps for absence of violence and sexual or illegal contents. The health-related application library of the National Health Service has a clinical assurance team to ensure that the topics provided by the health-related apps are all evidence-based and have no potential risk. However, they do not assess whether or not these apps can cause behavioral change [11].

On the other hand, computer and mobile games are highly popular. Games account for over one-third of the downloads [12]. For instance, 69% of British 8- to 74-year-olds play games averagely 14 h a week; out of which, 52% are females with a mean age of 31 years [13].

The idea of gamification was first documented in 2008, but its extensive application dates back to 2010 [14, 15]. Several definitions have been proposed for gamification; the most widely accepted definition is the “use of video game elements in non-gaming systems” [14]. The most commonly used gamification mechanisms include goals, rewards/scores, success badges, feedbacks, leaderboards, and any other factor that drives competition among users and enables social communication between them to encourage them to perform a favorable task or adopt a particular behavior. Many of these apps use a combination of these mechanisms. These elements make long-term behavioral change a pleasant experience for the users and prevent their discouragement [14, 15]. Gamification has several advantages. It improves the understanding of individuals regarding their capabilities, increases their occupational memory and concentration, and enhances their problem-solving capabilities and purpose-based function [15].

The existing inequalities in healthcare access, non-compliance to treatment and health instructions in long-term, and increased healthcare costs, as well as advances in digital equipment and availability of smartphones encouraged the healthcare providers to use gamification for their health-related purposes. Despite the popularity of gamification in personal computers, gamification of smartphone apps is a relatively new topic [14].

Evidence shows a correlation between gamification and behavioral change in the healthcare field [16]. According to review studies, gamification can positively affect health-related interventions and adoption of positive behaviors [17, 18]. In oral health field, some apps have used games to teach children a wide range of topics including tooth decay, healthy and carcinogenic diet.[19].

Dental caries is a common chronic disease of the childhood. A national report in Iran reported the caries index in 3 and 6-year-olds to be 1.9 and 5.2, respectively. Also, they reported that only 11% of 6-year-olds were caries-free, which is far from the target value of 90% stated by the World Health Organization [17].

Dental caries in childhood can be associated with severe pain and psychological problems, and may impose a high burden on the parents. Thus, dental associations worldwide recommend the first dental visit and initiation of toothbrushing at 1 year of age. However, parents often neglect this visit. Education via mobile apps can be a potential solution to enhance oral health knowledge of parents, particularly mothers, considering the popularity of mobile apps and their widespread use. Such apps can also be used to encourage healthy oral health-related behaviors. Parents are fully in charge of oral health status and oral hygiene of their preschool children and should supervise the oral hygiene behavior of their children as they grow up. To the best of the authors’ knowledge, no mobile app has been designed based on the principles of electronic designing of apps with educational content for oral health instruction of mothers. Limited apps have used gamification for this purpose; however, their efficacy has not been evaluated. Thus, this study sought to design a mobile app and assess the effect of gamification of app on quality of oral health instruction of mothers regarding oral healthcare of their children.

## Methods

This was a double-blind, parallel, pretest–posttest, controlled clinical trial with the allocation ratio of 1:1. The CONSORT statement is used as a guide to write this article [20]; see Additional file 2.

### Ethical considerations

Participation in the study was voluntary and the mothers signed informed consent forms prior to participation in the study. The study was approved by the ethics committee of School of Dentistry, Tehran University of Medical Sciences (IR.TUMS.VCR.REC.1397.1126). It was also registered in the Iranian Registry of Clinical Trials (IRCT20131102015238N2).

### Designing the app

The existing literature and the available media and mobile apps were evaluated aiming to find other apps in our field of research, inspiring designs for this app, and the required technical information to design it. Finally, we designed a simple app (without gamification) to promote oral-health knowledge of mothers using the best relevant evidence and guidelines according to the educational design principles. We have considered American Association for Pediatric Dentistry guidelines as well [21–23]. This app was designed for Android operating system with JAVA programming language in Android Studio version 3.1.4. The SQLite website was used to design and install the database, and DB Browser for SQLite version 3.10.1 was used to transfer data to the database. The designed app provided the mothers with oral healthcare information for their children such as oral hygiene, proper nutrition, fluoride intake, and dental visits. Also, the app could send a notification at 9 p.m. every night for the mothers reminding them to brush the teeth of their children. The app was evaluated by a group of oral medicine specialists, pediatric dentists and electronic learning and programming technicians, and the recommended modifications were made.

### Gamification of the app

Another version of this app was designed using the gamification elements. In the gamified version of the app, toothbrushing for the child, frequency of toothbrushing, application of toothpaste, and daily amount of intake of sugary substances by the child were the key elements reinforced by gamification; the mothers were asked questions about these topics and received a feedback for each response. She would be rewarded in case of giving the correct answer. The acquired scores for each question would be summed and the total daily score of the mother would be displayed. In case of acquiring a high score, the background color of the app would change for 1 week, indicating achieving a higher level. Also, a progress bar was present on the top of the page showing the scoring process for the purpose of encouragement. The opinion of the experts was asked regarding this app, and the modifications were made accordingly. Both apps were piloted on 2–3 mothers and their opinions after 1 week of using the app were collected and applied.

The app would send a notification to the mother at 9 p.m. every night. Clicking on the notification would redirect the user to the scoring section. The app was designed such that it would not allow accessing the scoring feature more than once daily. Also, the mother had to enter the child’s name and gender when signing up in the app. Thus, every time that the mother would log in, questions and notifications would include the name of her child, in order to be more user-friendly. Also, a female avatar would appear for baby girls and a male avatar would appear for baby boys on the home page of the app.

### Research environment and methodology

**Fig. 1:**
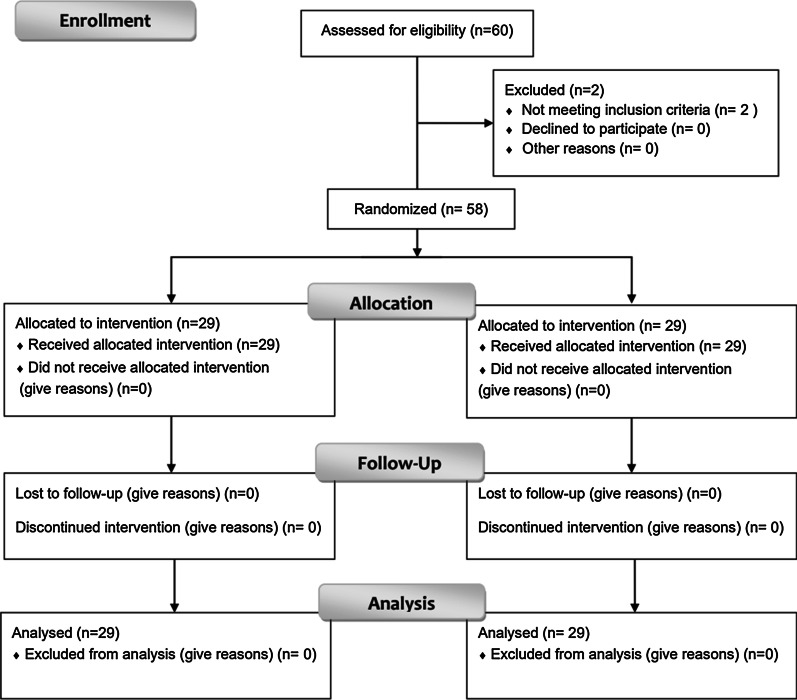
CONSORT flow diagram of the randomized controlled trial

In order to assess the efficacy of the apps, an intervention was performed on mothers of preschool children presenting to the specialty clinic of Tehran School of Dentistry. The collection data of our study was begun in March 2019 and ended by June 2019. The inclusion criteria of this study included the mother possessing a smart phone and her child being 6 years old or younger. The mothers were randomly divided into two groups (Fig. 1), to use either simple app or the gamified app, using a simple randomization method done by a computer software (Microsoft Excel).

The random allocation sequences were done by MZ, the enrolment of participants and their assignments to interventions were done by SZM, and they were examined by MSH.

The mothers in both groups filled out a questionnaire prior to downloading the app. The questionnaire (Additional file 1) assessed the oral health knowledge of mothers (18 questions) and their self-reported practice regarding oral health of their children (5 questions). It also had a demographic section regarding level of education of mother, level of education of father, socioeconomic status of the family, child’s age, and mother’s age. Also, the plaque index (PI) of children was measured and recorded in the questionnaire. PI was measured using a dental explorer and a dental mirror. MSH examined the children according to the “Leo & Silness” modified dental plaque index [24].

If the tooth had no plaque, it was given score zero, plaque present at gingival margin only, score one, and abundant dental plaque covering more than gingival margin, score two. For each child, the average level of plaque for all teeth scores was taken into account.

The questionnaire was designed using the available valid questionnaires [25–28] and included a number of researcher-designed questions too. The content and face validity of the questionnaire was assessed by a group of 7 experts of community oral health and pediatric dentistry. All item Content Validity Indexes were more than 0.83. Moreover, 10 mothers were requested to fill out the questionnaire twice with a 2-week interval to assess its reliability; the coefficient of agreement for all questions was at least 0.85.

Mothers who were considered in the assessment of the reliability of the study were excluded from the main study. Mothers were first interviewed with the questionnaire and after that the PI of the children was measured and recorded in a form at the end of the questionnaire.

Next, the apps were downloaded on mobile phones of the mothers. The address bar of the app had customer service contact information, and the mothers were asked to contact the customer service in case of having a problem.

After 1 month, the mothers were contacted by phone and were requested to show up for free fluoride therapy. The same questionnaire was filled out again by the mothers and the PI of their children was measured again.

### Sample size

Sample size was calculated to be 29 in each group considering the standard deviation of 2 for PI, study power of 80%, alpha = 5% and detection of 1.5 score difference between the two groups using the formula below:

$$n = \frac{{\left( {Z_{1 - \alpha /2} + Z_{1 - \beta } } \right)^{2} \left( {S_{1}^{2} + S_{2}^{2} } \right)}}{{\left( {\mu_{1} - \mu_{2} } \right)^{2} }}$$

### Blinding

Participants were blind in the current study. We had not informed participants that there were two different apps; however, the participants inevitably knew whether the app there were using is conventional or gamified. In accordance, the examiner was blind as well.

### Statistical analysis

Data were analyzed using PASW Statistics (Version 18). Descriptive data including percentage, mean and standard deviation were reported. The response to knowledge questions was dichotomized as correct and incorrect. Each correct answer was scored 1 and incorrect answers were scored 0. The maximum and minimum scores were 18 and 0, respectively. Regarding the five practice questions, the maximum and minimum scores that could be acquired were 14 and 0, respectively. Paired t-test was used to compare the pretest and posttest scores. The linear regression by the backward method was applied to assess the effect of demographic factors and type of intervention on the results.

## Results

In total, 60 pairs of mother and child were assessed for eligibility criteria and among those, 58 pairs had met the inclusion criteria. Next, mothers were randomly divided into two groups to use either the simple app (n = 29) or the gamified app, also take note that all participants had completed the study. Of all participants, 40% were boys and 60% were girls (Table 1). The majority of mothers had Bachelor’s degree while the majority of fathers had Master’s degree. Most mothers reported that they had good or moderate socioeconomic status. The mean age of mothers was 36 years in both groups and the mean age of children was 4.6 years. Our result revealed that 48.3% of children of simple-app group had a regular dental visit every six to one years, while only 37.9% of children of the gamified-app group had such dental visits. Moreover, 48.3% of mothers who were given the simple app and 72.4% of mothers who were given the gamified app used their app regularly.

**Table 1:** Demographic information of mothers and children in simple and gamified groups

Variable	Category	Simple app groups		Gamified app groups	
		Number	Percentage	Number	Percentage
Gender of the child	Boy	12	41.4	11	37.9
	Girl	17	58.6	18	62.1
Level of education of mother	Below high school diploma	0	0	1	3.4
	High school diploma	2	6.9	4	13.8
	College degree	5	17.2	4	13.8
	Bachelor’s degree	12	41.4	15	51.7
	Master’s degree	5	17.2	3	10.3
	Doctorate degree	5	17.2	2	6.9
Level of education of father	Below high school diploma	0	0	1	3.4
	High school diploma	1	3.4	4	13.8
	College degree	0	0	1	3.4
	Bachelor’s degree	2	6.9	6	20.7
	Master’s degree	22	75.9	13	44.8
	Doctorate degree	4	13.8	4	13.8
Child’s dental visit	When there is a problem	3	10.3	11	37.9
	Regularly every 6 months to once a year	14	48.3	11	37.9
	Irregular	5	17.2	3	10.3
	Etc. (explain…)	7	24.1	4	13.8
How did you use the application?	Seldom	7	24.1	1	3.4
	If needed	8	27.6	7	24.1
	Regular	14	48.3	21	72.4
Socioeconomic status of the family	Excellent	1	3.4	0	0
	Good	16	55.2	13	44.8
	Moderate	12	41.4	14	48.3
	Poor	0	0	2	6.9
Age of mother	Average	36.5		36.3	
	Standard deviation	4.9		4.5	
Age of child	Average	4.7		4.5	
	Standard deviation	1.3		1.1	

### Knowledge score of mothers before and after the intervention

In the pre-test, 100% of mothers in the simple app and gamified app groups gave a correct answer to 3 questions (questions 14, 15 and 16 in simple app group, and questions 13, 14 and 16 in gamified app group). In the post-test, 100% of mothers in the simple app group gave a correct answer to 7 questions (questions 1, 5, 6, 13, 14, 15, 16) and 100% of mothers in the gamified app group gave a correct answer to 4 questions (questions 5, 14, 15, 16). Note that the response «I don’t know» was considered as incorrect too. The maximum total knowledge score that could be acquired was 18. The mean total knowledge score acquired by mothers in the simple app group in the pretest was 10.5; this value was 11.3 in the gamified app groups. These values increased to 13.1 and 14.3, respectively in the posttest (Table 2).

**Table 2:** The mean total knowledge score acquired by mothers before/after intervention in simple and gamified groups

Group	Before the intervention		After the intervention		*P* value
	Average	standard deviation	Average	standard deviation	
Simple app group	10.5	2.1	13.1	1.6	< 0.001
Gamified app group	11.3	1.9	14.3	2.0	< 0.001

### Practice of mothers regarding oral health of their children before and after the intervention

All children had started toothbrushing and about half of them brush their teeth by themselves. The maximum total practice score that could be acquired was 14. The mean total practice score of mothers regarding oral health of their children in pretest was 4.4 in the simple app and 4.8 in the gamified app group. These values were 8.5 and 8 in the posttest, respectively (Table 3).

**Table 3:** The mean total practice score acquired by mothers before/after intervention in simple and gamified groups

Group	Before the intervention		After the intervention		*P* value
	Average	Standard deviation	Average	Standard deviation	
Simple app group	4.4	2.4	8.5	1.7	< 0.001
Gamified app group	4.8	3.2	8	2.2	< 0.001

### PI of children before and after the intervention

The PI of children before the intervention was 0.8 and 1 in the simple app and gamified app groups, respectively; these values decreased to 0.5 and 0.5, respectively after the intervention (Table 4).

**Table 4:** Plaque index of children before/after intervention in simple and gamified groups

Group	Before the intervention		After the intervention		*P* value
	Average	Standard deviation	Average	Standard deviation	
Simple app group	0.8	0.4	0.5	0.3	0.006
Gamified app group	1	0.3	0.5	0.3	< 0.001

### Effect of demographic factors and type of intervention on oral health knowledge and practice of mothers and PI of their children

There were no association between demographics and type of intervention on knowledge and practice of mothers of their children.

The change in practice of mothers regarding PI of their children (determined by clinical examination) was greater in the gamified app group compared with the simple app group (B = − 0.210, *P* = 0.038).

## Discussion

This study assessed the effect of gamification of a designed oral healthcare app on the knowledge and practice of mothers regarding oral healthcare of their children. We designed an app for this purpose since smartphone apps are gaining increasing popularity worldwide. We assessed and compared the efficacy of a simple app and the gamified version of this app. The results showed enhancement of oral healthcare knowledge and self-reported practice of mothers in both groups. However, the children of mothers in the gamified app group showed a greater reduction in PI compared with the simple app group. Since the clinical outcome was the primary outcome measure in our study, this finding highlights the optimal effect of gamification of the app on the outcome.

Early childhood caries is common in Iran and around 50% of 3-year-olds and 90% of 6-year-olds have early childhood caries [17]. Mouradian [29] evaluated dental education and oral health of children and reported that children at higher risk of dental problems had less access to healthcare services. This indicates poor education at the level of dental clinicians and also caregivers of children i.e. mothers. Oral health of children is an ethical responsibility shared by all community healthcare providers [29]. Mothers play a fundamental role in this respect and parents are fully responsible for oral health care of their preschool children. Also, evidence shows that children with higher sugar intake under the supervision of mothers keep this habit in older ages as well [30].

An effective health instruction should enhance knowledge, create positive attitude, change belief, enhance skill acquisition, and cause behavioral or life style changes. Reinforcement of a particular behavior by external motivations such as notifications can enhance the process of behavioral change.

The knowledge score of mothers in our study was moderate (knowledge score of 10 or 11 out of a maximum of 18). Their practice score was poor. Also, half of children brush their teeth by themselves, which is against the protocol by the World Health Organization for oral hygiene in preschoolers, and indicated the need for instruction of mothers in this respect. Preschoolers usually do not have a regular dental visit in Iran, and the first visits occur between the ages of 3 and 6 [31]. This emphasizes the need for virtual instruction of mothers on this topic. Evidence shows that people are interested to have their health-related information on their smartphones, and over 500 million people worldwide use health-related mobile apps [6]. Smartphones can be an effective tool for provision of oral health information, causing oral health behavioral change and improving oral hygiene. Thus, we designed a mobile app, which did not require a strong Internet connection. We primarily searched the web and found some mobile apps, which were all in English. They all had a simple function and could only provide simple information. They had been mostly designed according to the preferences of the users and not based on the opinion of the experts. Thus, they did not seem to be highly effective for behavioral change [7–10]. A study conducted in Saudi Arabia designed an app to enhance the knowledge of mothers regarding health status of their children; however, they did not perform any clinical examination [32]. Another study conducted in the United States reported that most designed apps did not address the oral disease risk factors such as diet, alcohol consumption, and tobacco use. In general, these apps had a poor performance regarding creation of motivation, and generally had a poor quality [33].

A study conducted in Brazil reported that most mobile phone apps had not been certified by any dental organization. They recommended that such apps should provide the most recent evidence-based information and should be periodically monitored in terms of quality [34].

In the present study, we designed an app according to the American Association for Pediatric Dentistry guidelines and assessed its reliability. Also, we assessed the validity and reliability of our data collection tool based on the most recent scientific evidence.

A systematic review conducted in 2018 reported that behavioral change mechanisms such as feedback and monitoring had been used in 60 (94%) software programs, reward and threat had been used in 52 (81%), and goals and planning had also been used in 52 (81%) programs. The most common individual techniques used in combination with each other included goals, personal monitoring, rewards and non-specific encouragement (55%). They concluded that limited health-related software programs had used gamification and called for further studies on the efficacy of behavioral change techniques and clinical outcomes [14]. Inspecting our results, the number of mothers who used the application regularly was higher in the game group compare to simple group; this could be thanks to the playful structure of gamified app.

The app designed in our study was gamified using feedback and monitoring, rewards, badges, scores, and leveling to create, reinforce and stabilize a behavior. In our study, knowledge and self-reported practice of mothers and PI of their children improved in both groups of simple and gamified apps after the intervention compared with baseline. Regarding the oral-health knowledge, the questions that both groups have answered correctly were in two areas: one question regarding the time to start cleaning a child's teeth, and several questions about cariogenic foods. It is worth remarking that all mothers learned about cariogenic substances, as well as proper time to start cleaning their children's teeth. This is particularly relevant for the prevention of caries. In the gamefied-app group, one of the questions that all the mothers had answered correctly in the pre-test was not anymore 100% correctly answered in the post-test. The reason of this change might be that some mothers had doubted about their answers the second time they were asked the same questions, and hence had chosen the option «I don't know». To sum up, the repetition of correct answers in questionnaire was not one hundred percent. Most previous studies on the efficacy of simple and gamified apps did not assess the clinical outcome. However, in this study, we measured the PI of children to assess the clinical outcome. When recalling the mothers, we did not mention anything about clinical examination. Also, they were asked to show up for fluoride varnish therapy (which would not require prior toothbrushing). This was done to prevent exaggeration of plaque control. The results showed that the gamified app was more effective for PI change of children than the simple app, which highlights the optimal efficacy of gamification in achieving the desired clinical outcome. It seems that rewarding, success badges, feedbacks, leaderboards and any other factor that would show an individual’s progression are effective for encouraging a favorable behavior and prevent discouragement [14, 15]. We have, however, measured the plaque index, which is a short-term clinical consequence of the intervention hoping that this would result in more favorable health outcome as well. Future studies may examine the effect of applications on caries, as an ultimate long-term consequence of intervention.

A review study on the efficacy of gamification for self-care in diabetic patients showed that gamification created a positive reinforcement as an external motivation and was effective for self-care promotion in diabetic patients [35]. Another study conducted in the Netherlands showed that people who used a gamified physical exercise app were physically more active and spent more time outside the house. They concluded that gamification in healthcare interventions can positively affect behavioral change [36]. The current results were in agreement with theirs since greater behavioral change was achieved in the gamified app group.

This study does not include questions regarding previous participation to oral-health educational programs as they are not so common for preschoolers in Iran. Since we randomly divided the participants into two groups, we expect the random allocation to eliminate the effect caused by not asking this question. Moreover, in this study, due to cultural issues we have preferred to use indirect income indicators; one may use direct indicators such as monthly income in future studies when possible.

## Conclusion

Health behaviors are established during childhood and mothers play a key role in this respect. The mothers evaluated in our study did not have adequate knowledge or practice regarding oral healthcare of their children at baseline. Both apps effectively promoted the oral health of their children. However, reduction of PI, as the main cause of dental caries, was greater in children of mothers who used the gamified app.

### Suggestions

Considering the optimal efficacy of the designed app, it should be marketed for its widespread use by the healthcare system. There were some bugs in notifications in more recent versions of smartphones, which need to be addressed in the updates of the app. Also, future studies are recommended to focus on the oral hygiene of mothers since their oral hygiene practice affects their children as well.

## Supplementary information

**Additional file 1.** Questionnaire about mother’s oral-health knowledge, mother’s practice about children, demographic data and child’s clinical examination.

**Additional file 2.** CONSORT checklist: Completed CONSORT checklist for our randomized trial study.

## Data Availability

The datasets used and analyzed during the current study are available from the corresponding author on reasonable request.

## Abbreviations

App: Application
PI: Plaque index
