# Supplementary material for: Development and evaluation of a gamified smart phone mobile health application for oral health promotion in early childhood: a randomized controlled trial
Source: BMC Oral Health. 2021 Jan 7;21:18. doi: 10.1186/s12903-020-01374-2 (PMC7791794; doi:10.1186/s12903-020-01374-2)
Supplement: Supplementary file 1 — Additional file 1. Questionnaire about mother’s oral-health knowledge, mother’s practice about children, demographic data and child’s clinical examination. [file 12903_2020_1374_MOESM1_ESM.pdf]

Questionnaire about mother's oral-health knowledge, mother's practice about children, demographic data and child's clinical examination:

Knowledge questions:

1.1 . The first primary tooth erupts averagely at 6 months of age.

Yes ☐ No ☐ I do not know ☐

1.2. Bacteria responsible for dental caries often transfer from mother to child.

Yes ☐ No ☐ I do not know ☐

1.3. Fluoridated toothpastes should not be used for children under 3 years of age.

Yes ☐ No ☐ I do not know ☐

1.4. White lines or spots are the first signs of caries in children.

Yes ☐ No ☐ I do not know ☐

1.5. Tooth cleaning and toothbrushing should be started following the eruption of the first primary tooth.

Yes ☐ No ☐ I do not know ☐

1.6. Fluoridated toothpastes prevent dental caries.

Yes ☐ No ☐ I do not know ☐

1.7. Children should not use fluoride.

Yes ☐ No ☐ I do not know ☐

1.8. Frequency of intake of sugary substances plays a more important role than the total amount of consumption of sugar in caries development.

Yes ☐ No ☐ I do not know ☐

1.9. Irrigation of teeth with copious water after toothbrushing can effectively prevent caries.

Yes ☐ No ☐ I do not know ☐

1.10. Consumption of xylitol in the form of chewing gum or snacks can decrease dental caries.

Yes ☐ No ☐ I do not know ☐

1.11. The acidity of oral environment caused by the activity of bacteria after meals returns to normal after 5 min.

Yes ☐ No ☐ I do not know ☐

1.12. Bottle-feeding can cause early childhood caries.

Yes ☐ No ☐ I do not know ☐

1.13. Carious primary teeth need to be restored.

Yes ☐ No ☐ I do not know ☐

The following items can cause dental caries.

Item	Yes	No	I do not know
1.14 Candy			
1.15 Fruit juice			
1.16 Cola			
1.17 Cheese			
1.18 Tea			

Practice questions:

2.1. Who brushes the teeth of your child?

- ☐ He/she does not brush yet
- ☐ Himself/Herself
- ☐ My other child
- ☐ Himself/Herself under the supervision of adults
- ☐ Adults

2.2. How often does your child brush his/her teeth?

- ☐ Irregularly or never
- ☐ Once a week
- ☐ Several times (2-3 times) a week
- ☐ Once a day
- ☐ More than once a day

2.3. Does your child rinse his/her mouth after eating sugary foods?

- ☐ Always
- ☐ Most of the time
- ☐ Occasionally
- ☐ Never

2.4. How often does your child have a sweet snack or sweet drink (warm or cold)?

- ☐ Three times or more per day
- ☐ Around 2 times per day
- ☐ Once a day
- ☐ Occasionally, not everyday
- ☐ Rarely

2.5. Do you use fluoridated toothpaste for your child?

- ☐ Always
- ☐ Most of the time
- ☐ Occasionally
- ☐ Never
- ☐ I do not know

Demographic questions:

Gender of the child?

Age of the child?

Age of mother?

Level of education of mother?

- ☐ Below high school diploma
- ☐ High school diploma
- ☐ College degree
- ☐ Bachelor’s degree
- ☐ Master’s degree
- ☐ Doctorate degree

Level of education of father?

- ☐ Below high school diploma
- ☐ High school diploma
- ☐ College degree
- ☐ Bachelor’s degree
- ☐ Master’s degree

- [ ] Doctorate degree

Socioeconomic status of the family?

- [ ] Poor
- [ ] Moderate
- [ ] Good
- [ ] Excellent

How often does your child have a dental visit?

- [ ] When there is a problem
- [ ] Regularly every six months to once a year
- [ ] Irregular
- [ ] Etc. (explain…)

How often did you use the application?

- [ ] Seldom
- [ ] If needed
- [ ] Regular

Phone number:

Clinical examination:

Plaque index (maximum score for mesiobuccal, midbuccal, distobuccal, and midlingual surfaces recorded for each tooth)

0: No dental plaque

1: Slight amount of dental plaque

2: High amount of dental plaque

3: Unerupted tooth

55	54	53	52	51		61	62	63	64	65
85	84	83	82	81		71	72	73	74	75
